# Stepwise Synthesis of Au@CdS-CdS Nanoflowers and Their Enhanced Photocatalytic Properties

**DOI:** 10.1186/s11671-019-2977-z

**Published:** 2019-04-29

**Authors:** Liwei Wang, Zhe Liu, Junhe Han, Ruoping Li, Mingju Huang

**Affiliations:** School of Physics and Electronics, Henan University, Kaifeng, 475004 People’s Republic of China

**Keywords:** Hybrid nanostructure, Charge transfer, Visible-light photocatalyst, Complex morphology, Epitaxial growth

## Abstract

Fabrication of hybrid nanostructures with complex morphologies and high photocatalytic activity is a difficult challenge because these particles require extremely high preparation skills and are not always practical. Here, hierarchical flower-like Au@CdS-CdS nanoparticles (Au@CdS-CdS nanoflowers) have been synthesized using a stepwise method. The Au@CdS-CdS nanoflowers are consisted of Au core, CdS shell, and CdS nanorods. The UV-Vis absorption range of the Au@CdS-CdS nanoflowers reaches up to 850 nm which covers the whole visible range (400–760 nm). Photoinduced charge transfer property of Au@CdS-CdS nanoflowers was demonstrated using photoluminescence (PL) spectroscopy. Compared to CdS counterparts and Au@CdS counterparts, Au@CdS-CdS nanoflowers demonstrated the highest photocatalytic degradation rate under irradiation of λ = 400–780 nm and λ = 600–780 nm, respectively. Based on structure and morphology analyses, we have proposed a possible formation mechanism of the hybrid nanostructure which can be used to guide the design of other metal-semiconductor nanostructures with complex morphologies.

**Electronic supplementary material:**

The online version of this article (10.1186/s11671-019-2977-z) contains supplementary material, which is available to authorized users.

## Introduction

Solar energy is currently recognized as the most clean and abundant energy source. The conversion of solar energy into other energy forms using semiconductor photocatalyst has been regarded as an ideal way to solve the energy crisis and the pollution of the environment [1, 2]. To make full use of solar energy, many semiconductor-based photocatalysts have been designed and developed [3, 4]. Among them, CdS, with a suitable band gap about 2.4 eV for effective visible light absorption of sunlight, is one of the few visible light-driven photocatalysts [5]. However, the high charge carrier recombination rate and photocorrosion seriously impede the photocatalytic efficiency of CdS.

In recent years, hybrid nanostructures have attracted extensive attention due to their physicochemical properties [6–9]. Compared with the corresponding individual component, CdS-based hybrid nanostructures show better performance in certain application due to the enhanced light absorption range and improved the charge separation. Yu et al. have constructed a unique ternary sulfide -[ZnS-CdS-Cu_2-x_S]-ZnS- heteronanorod which shows an expanded absorption region and efficient charge separation, leading to an improved performance in solar energy conversion [10]. Xu et al. have synthesized spherical- and rod-shaped CdS@TiO_2_ core-shell nanoparticles (CSNs) which demonstrate excellent photocatalytic performance for selective redox reactions under visible-light irradiation [11, 12]. The Z-scheme in CdS-Au-TiO_2_ three-component nanojunction system exhibited higher photocatalytic activity than those of single- and two-component systems, due to the transfer of electrons along the designed route [13]. Although many CdS-based hybrid nanostructures have been synthesized, the controlled synthesis of hybrid nanostructures with desired components, structures, and crystal phases remains a notable challenge.

As an important branch of hybrid nanostructures, metal-semiconductor nanostructures have been widely studied as a new type of advanced functional material [14–19]. Among them, Au-CdS hybrid nanostructures have attracted increasing attention due to the interaction between plasmon and exciton. Plasmonic nanostructures have significant abilities to concentrate light energy at the nanoscale, which comes from their nature as a collective oscillation of surface electrons of the metal with incident light at matched frequencies [20]. When metals are hybridized with semiconductors, such nanoscale light managing ability of plasmonics could be utilized to improve the efficiency of photoexcitation in the semiconductors [21]. In nanoscience research, designing complex nanostructures by controlling the size and shape of nanocrystals is critical to exploring their novel properties [22–25]. So far, various shapes of Au-CdS hybrid nanostructures have been prepared and applied to optical components, sensors, photovoltaic, and photocatalytic devices [26–30]. However, 3D hierarchical structure of Au-CdS nanocomposites with well-defined morphology have rarely been reported. The synthesis of complex nanocomposites is a very difficult challenge because these nanoparticles require extremely high preparation skills and are not always practical.

Herein, we constructe a flower-like Au@CdS-CdS nanoparticles (Au@CdS-CdS nanoflowers) through a stepwise synthesis strategy. Which has hierarchical heterostructures constructed by epitaxial growth of 1D CdS nanorods on the surfaces of Au@CdS CSNs and heterogeneous structure between Au and CdS. The synthesized Au@CdS-CdS nanoflowers show an expanded absorption region which reaches up to 850 nm and cover the whole visible range. Au@CdS-CdS nanoflowers demonstrate enhanced photocatalytic activity compared to Au@CdS CSNs and CdS counterparts under the visible irradiation. Based on its size, morphology, and structure, we have proposed a feasible growth mechanism, which probably be useful to researchers in areas as diverse as medicine, energy, and electronics who often design complex nanoparticles that are predicted to have useful functions.

## Methods

### Synthesis of Au@CdS-CdS Nanoflowers

All chemicals received from Aladdin were analytic grade reagents and used without further purification. Au colloids with an average diameter of 50 nm were synthesized by traditional Frens’ method [31] and Au@CdS CSNs were prepared using a published hydrothermal method [32]. Typically, an aqueous solution of l-cysteine (Cys, 99%) was mixed with cadmium nitrate tetrahydrate (99%) in a 2:1 M ratio of Cys to Cd^2+^. 2 mL l-cysteine-Cd^2+^ (Cys/Cd^2+^) mixture were added into 10 mL Au colloids and stirred for 15 min, then the obtained Au-(Cys/Cd^2+^) were diluted to 50 mL with water and transferred into a Teflon-lined stainless-steel autoclave (100 mL capacity). After sealed, the autoclave was heated and maintained at 130 °C for 6 h. To coat thick CdS shell on Au nanoparticles, 10 mL Cys/Cd^2+^ mixture were added into Au colloids (10 mL) with the other reaction conditions unchanged.

Au@CdS-CdS nanoflowers were then synthesized as follows: the resulting Au@CdS CSNs colloids (10 mL), 1 mL of 10 mM Cys/Cd^2+^ mixture and 20 mL ethylenediamine (En, > 99%) were mixed and stirred for 15 min, and then transferred into a Teflon-lined stainless-steel autoclave (50 mL capacity). The sealed autoclave was heated to 180 °C and maintained at this temperature for 10 h, and then allowed to cool down to room temperature. The product was collected and washed with distilled water and ethanol several times to remove remaining ions and impurities.

CdS counterparts were synthesized as follows: 4 mL Cys/Cd^2+^ mixture and 20 mL water were added to the reactor and stirred for 15 min, then sealed and heated to 130 °C for 6 h. The products were cooled naturally down to room temperature and washed with distilled water and ethanol several times. Finally, the samples were dispersed in absolute ethanol.

### Material Characterizations

The X-ray diffraction (XRD) measurements of the samples were performed on a Bruker D8 advanced diffractometer and the UV-Vis absorption spectra were tested on a PerkinElmer Lambda 35 photometer. Field emission scanning electron microscope (FE-SEM) images and energy dispersive X-ray spectrometer (EDS) were obtained on a JSM-7001F device. The transmission electron microscope (TEM) model is JEM-2010 and the operating voltage is 200 kV. High-resolution transmission electron microscope (HR-TEM) images were also tested on JEM-2010 with an operating voltage of 200 kV. Photoluminescence (PL) spectra were measured on a Perkin-Elmer LS-55 with an excitation wavelength of 400 nm. Photocurrent measurements (i–t curves) were conducted in a conventional three-electrode cell system by using a CHI660C electrochemical station without bias under a 300 W Xe arc lamp as light source. The cleaned ITO glass deposited with photocatalysts, Pt flake, and an Ag/AgCl electrode were, respectively, used as working electrodes, counter electrode, and reference electrode. Na_2_SO_4_ (0.2 M) was used as the electrolyte.

### Photocatalytic Activity Measurement

The optical system used for photocatalytic reaction consisted of xenon lamp (300 W) and two bandpass filters (with the bandwidth of 400–780 nm and 600–780 nm) that ensure the irradiation in visible range. Four kinds of photocatalysts including Au nanoparticles, CdS counterpart, Au@CdS thick, and Au@CdS-CdS nanoflowers were used to degrade rhodamine 6G (R6G) solution. Typically, 6 mg photocatalyst was added into 20 mL R6G solution (1.0 × 10^−5^ M) and stirred for 30 min in the dark to achieve adsorption equilibrium before irradiation. The photocatalytic experiments were conducted under two irradiation range, 400–780 nm and 600–780 nm, respectively. Then, 2.5 mL solution were extracted every 10 min and centrifuged to remove the photocatalyst. The UV-Vis absorption spectra of filtrates were recorded using PerkinElmer Lambda 35 photometer to monitor the catalytic reaction. All the photocatalysis experiments were conducted at room temperature in air. Degradation of R6G was defined as follows:

Degradation (%) = [(*C*_0_-*C*_t_) / *C*_0_] × 100.

where *C*_0_ is the initial concentration of R6G, *C*_t_ is the concentration of R6G at a certain time after the photocatalytic reaction.

## Results and Discussion

### The Synthesis Mechanism of Nanoflowers

**Fig. 1:**
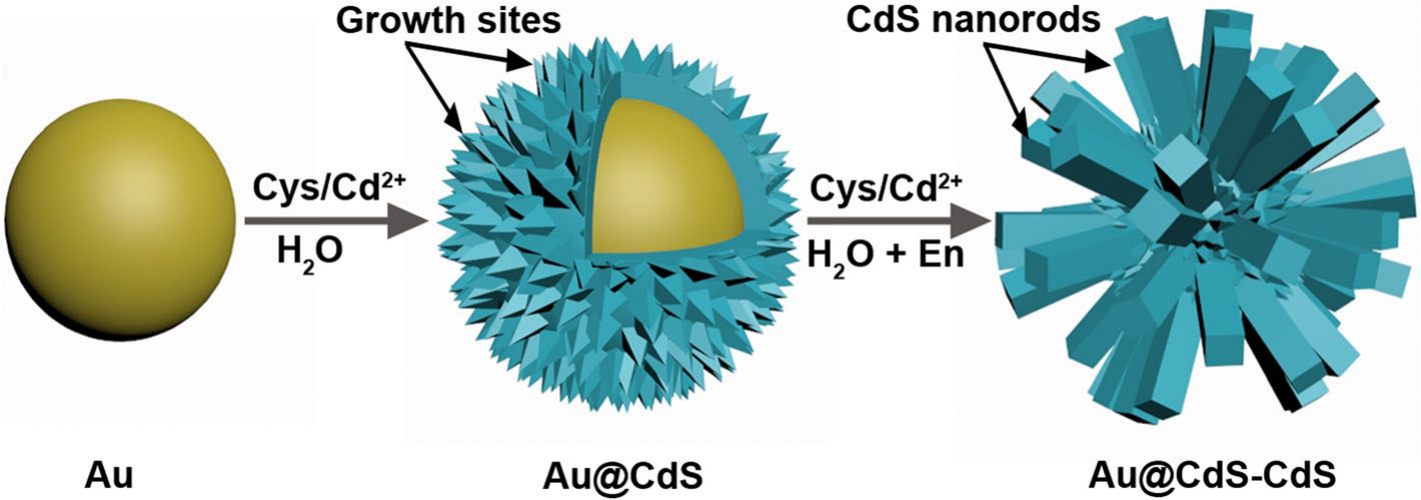
Schematic illustration of the synthesis processes of Au@CdS-CdS nanoflowers

The stepwise synthesis process for the Au@CdS-CdS nanoflowers is depicted in Fig. 1. First, Au nanoparticles are synthesized with an average size about 50 nm. The CdS layer is deposited on the surface of the Au nanoparticles through a hydrothermal reaction to produce Au@CdS CSNs. Then, the CdS nanorods are grown on the surface of Au@CdS CSNs through a mixed solvothermal method to produce the hierarchical Au@CdS-CdS nanostructures. l-cysteine and ethylenediamine play an important role in the synthesis of Au@CdS-CdS nanoflowers. l-cysteine, possessing three functional groups (SH, COOH, NH_2_), serves both as a sulfur source and linking agent to make up for the lattice mismatch between Au and CdS crystals [32]. The crystal phase could be controlled by introducing ethylenediamine as a structure-guiding reagent in the growth of CdS nanorods [33]. In addition, it is very difficult for us to synthesize Au@CdS-CdS nanoflowers by adding Au colloids, Cys/Cd^2+^ mixture, and ethylenediamine together under the same conditions. Therefore, the intermediate of Au@CdS CSNs is necessary for the synthesis of Au@CdS-CdS nanoflowers, because the surface of the CdS shell is not smooth and these bulges become the “growth sites” for the epitaxial growth of CdS nanorods.

### Sample Morphology and Structure

**Fig. 2:**
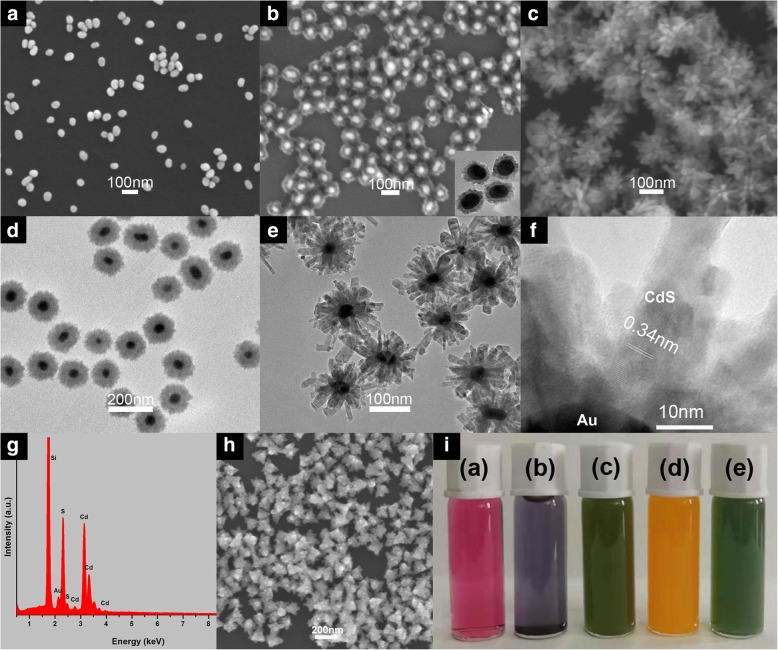
**a**, **b**, **h** SEM images of Au nanoparticles, Au@CdS with thin shell, and CdS counterparts, respectively. **d** TEM images of Au@CdS with thick shell. **c**, **e**, **f**, **g** SEM, TEM, HR-TEM image, and EDS profile of Au@CdS-CdS nanoflowers. **i** Photographs of their solution colors

The SEM images of the nanoparticles in each procedure were shown in Fig. 2a–c. Au nanoparticles showed that they were uniform in size and well dispersed. Au@CdS CSNs were shown in Fig. 2b in which single Au or CdS nanoparticles were rarely observed. The surface of CdS shell was serrated and the bulges could be observed in the insert TEM image. As exhibited in Fig. 2c, the Au@CdS-CdS nanocrystals presented as well-defined flower-like nanoparticles with well dispersity. Figure 2d showed the TEM image of Au@CdS CSNs with thick CdS shell (denoted as Au@CdS thick). Figure 2e showed the TEM image of Au@CdS-CdS nanoflowers. It can be seen that 1D CdS nanorods were epitaxial grown on the surface of Au@CdS CSNs. The diameter and the length of the CdS nanorod was about 16 nm and 40 nm, respectively. The HR-TEM image of Au@CdS-CdS nanoflowers were shown in Fig. 2f. The lattice spacing of 0.34 nm in nanorods was consistent with the (002) crystal planes of wurtzite CdS. The as-prepared CdS nanorods were single crystals with a preferential growth direction of [001], the c-axis [34]. The EDS analysis further confirmed the elemental composition of the Au@CdS-CdS nanoflowers, as shown in Fig. 2g, which indicated the presence of the elements of Cd, S, and Au. CdS nanocrystals with uniform size were shown in Fig. 2h. In addition, the colors of the solution of Au colloids, Au@CdS thin, Au@CdS-CdS nanoflowers, CdS counterparts, and Au@CdS thick were red wine, purple, green, yellow, and green, respectively, shown in Fig. 2i.

**Fig. 3:**
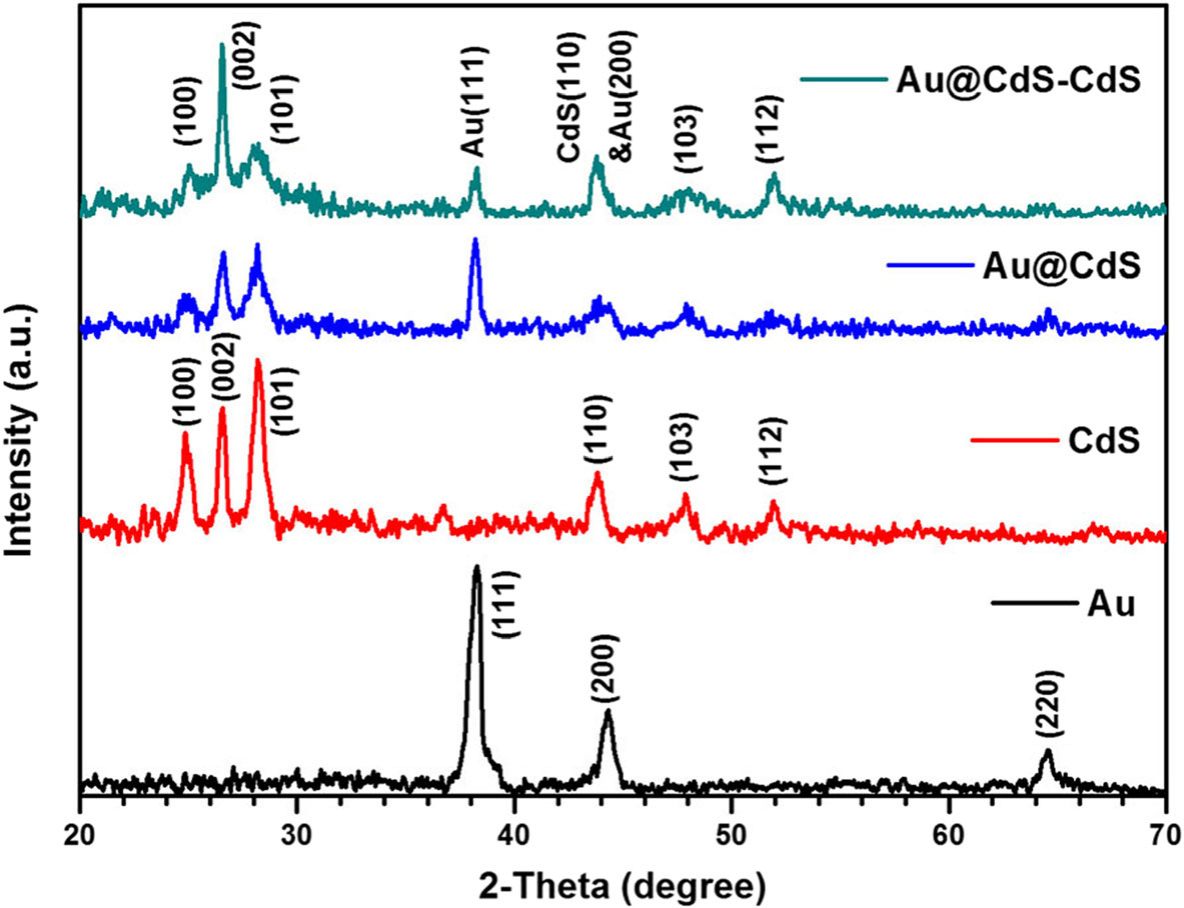
XRD patterns of Au, CdS, Au@CdS CSNs, and Au@CdS-CdS nanoflowers

The XRD patterns of the samples obtained in the synthesis process of Au@CdS-CdS nanaoflowers were shown in Fig. 3. XRD peaks of Au nanoparticles was observed and the peaks at 2θ = 38.2, 44.2, and 64.75 were due to the (111), (200), and (220) planes, respectively of the fcc Au crystal (JCPDS 04-0784). For CdS crystals, the peaks at 2θ = 25, 26.5, 28.2, 43.8, 47.8, and 51.9 were due to (100), (002), (101), (110), (103), and (112) planes of the wurtzite CdS crystal (JCPDS 41-1049). Both fcc Au and wurtzite CdS crystal diffraction peaks were observed in the XRD patterns of Au@CdS CSNs and Au@CdS-CdS nanoflowers, respectively. It should be noted that the diffraction peak intensity of the (002) crystal plane was far higher than other peaks in Au@CdS-CdS nanocrystals. This indicated that the diffraction peak of the (002) crystal plane is prominent and the CdS nanorods were grown along [001] direction, which was in accordance with the HR-TEM result.

### Optical Properties of the Sample

**Fig. 4:**
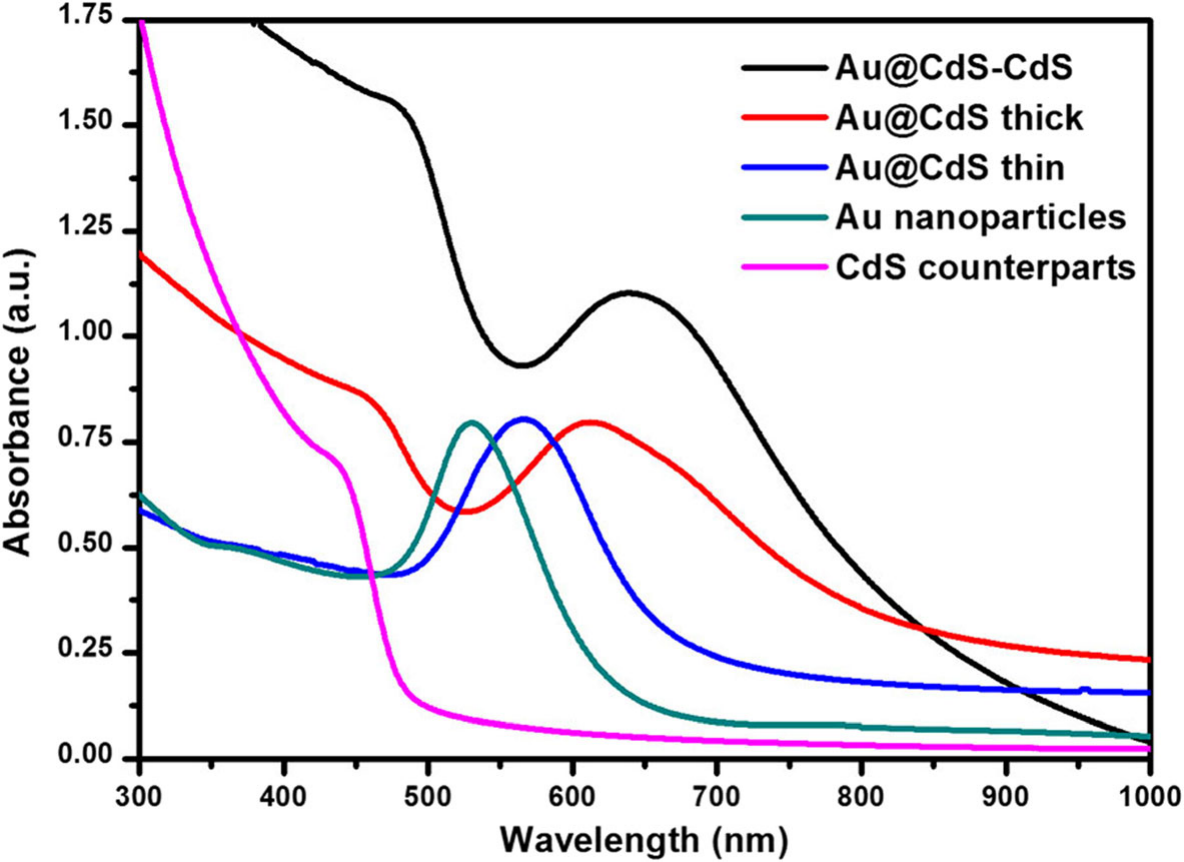
The UV-Vis absorption spectra of samples: CdS counterparts, Au nanoparticles, and Au@CdS CSNs and Au@CdS-CdS nanoflowers, respectively

The optical properties of the five samples were analyzed by UV-Vis measurements, as depicted in Fig. 4. The band edge position of CdS colloids was less than 500 nm. The 50 nm Au colloids exhibits a surface plasmon resonance (SPR) absorption centered at 530 nm. Coating the Au nanospheres with thin CdS layer resulted in a red-shift of the plasmon peak to 568 nm. With CdS shell thickness increase, the SPR position further red-shifted to 623 nm and 635 nm in the Au@CdS thick and Au@CdS-CdS nanoflowers, respectively. The experimental results showed that with the increase of the thickness of the CdS shell, the SPR absorption peak of the Au nanoparticles exhibited a continuous red-shift, because the SPR position of the metal depended on the metal size, shape, and surrounding media. Similar phenomena were observed in Au@SiO_2_, Au@Cu_2_O, and Au-Fe_3_O_4_ hybrid nanostructure systems [35–37]. The band edge position of Au@CdS-CdS nanoflowers reached up to 850 nm, which could better harvest the visible region of the sunlight (400–760 nm) to enhance photocatalytic activity.

### Light-Induced Charge Transfer Properties

**Fig. 5:**
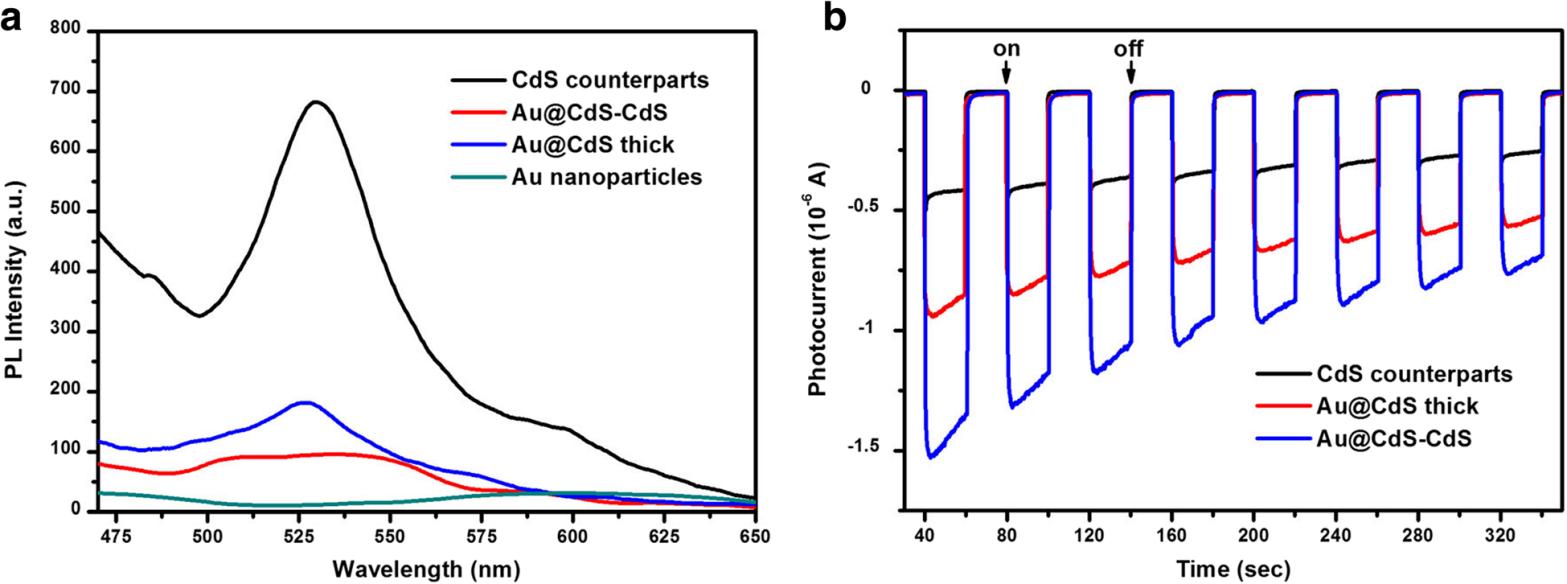
**a** The PL spectra of Au colloids, Au@CdS thick, Au@CdS-CdS, and CdS counterparts, respectively. **b** Comparison of transient photocurrent response of the Au@CdS-CdS nanoflowers, Au@CdS thick, and CdS counterparts irradiated with visible light of λ ≥ 400 nm in 0.2 M Na_2_SO_4_ aqueous solution without bias versus Ag/AgCl

The photoluminescence (PL) spectra of the synthesized Au@CdS-CdS nanoflowers, Au@CdS CSNs, CdS counterparts, and Au colloids were shown in Fig. 5a. The gold colloids had a weak PL peak at ~ 600 nm. The PL spectrum of the CdS counterparts showed a dominant emission band at 530 nm and a smaller shoulder peak at 598 nm. The band at 530 nm could be attributed to the typical excitonic band-band emission of CdS. The shoulder at 598 nm might be due to trapped emission which arose from structural defects in the CdS crystal. However, the PL spectral intensity of the hybrid structure were significantly reduced, and the radiative emission of the CdS in Au@CdS-CdS nanoflowers was quenched more than that in Au@CdS CSNs. Since the Fermi level of Au was located at + 0.5 V relative to the normalized hydrogen electrode (NHE), it was lower than the conduction band level of CdS (− 1.0 V vs. NHE). After CdS excited, the free electrons in the conduction band would be transferred to the Au core due to the energy difference [32, 38]. The photoinduced charge transfer mechanism led to a decrease in the electron-hole recombination probability and suppression of exciton emission in CdS. The recombination probability of photogenerated electron and hole was greater in the Au@CdS CSNs, probably due to the high content of CdS. Flower-like Au@CdS-CdS hybrid nanostructures provide an ideal research platform for the study of photoinduced charge transfer mechanism for metal-semiconductor heterojunction. The results of the transient photocurrent response obtained from Au@CdS-CdS nanoflowers, Au@CdS thick, and CdS counterparts were shown in Fig. 5b. It was easy to observe that the photocurrent over Au@CdS-CdS was greatly improved compared with that of Au@CdS thick and CdS counterparts. The enhanced photocurrent over the Au@CdS-CdS nanocomposite implied more efficient separation of the photoinduced electron-hole pairs and a longer lifetime of the photogenerated charge carriers than that of blank-CdS, which was beneficial for its photocatalytic activity.

### Application of Materials

**Fig. 6:**
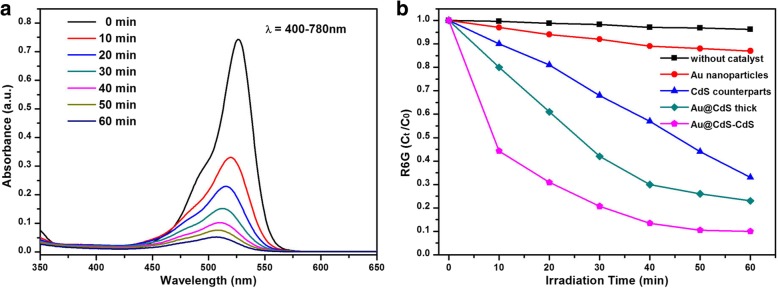
**a** Absorption spectra of R6G solutions at different irradiation times by using Au@CdS-CdS nanoflowers as photocatalyst under irradiation of 400–780 nm. **b** *C*_t_/*C*_0_ versus irradiation time plots for R6G photodegradation in the presence of different photocatalysts

Au@CdS-CdS nanoflowers have enhanced visible light trapping capability and photo-induced charge separation properties, which have potential value in certain application. The photocatalytic activity of Au@CdS-CdS under 400–780 nm irradiation was detected by using dye Rhodamine 6G as a photodegradation target. For comparison, Au nanoparticles, CdS counterparts, and Au@CdS thick were also tested under the same conditions. Figure 6a was the UV-Vis absorption spectra of the R6G solutions under different irradiation times by using Au@CdS-CdS nanoflowers. The intensity of the absorption peak of the R6G solution decreased rapidly with the illumination time, and its characteristic absorption peak also shifted from 528 nm to 507 nm. After 40 min, the concentration of R6G almost had no changed, suggesting that the degradation reaction was complete. Figure 6b showed the normalized change of the concentration of R6G solutions with Au nanoparticles, CdS counterparts, and Au@CdS thick and Au@CdS-CdS nanoflowers. Without catalyst, the change of concentration of R6G could be negligible. Among the catalysts, the photodegradation rate of Au@CdS-CdS nanoflowers was significantly faster than that of other materials. After 40 min, 86% of the R6G solution was degraded in the Au@CdS-CdS nanoflowers system, whereas only 71%, 46%, and 12% of the R6G solution was degraded in the Au@CdS thick, CdS counterparts, and Au nanoparticles system, respectively.

As seen in Fig. 6b, hybrid nanostructures exhibited superior photocatalytic activity compared with corresponding single-component ones. Embedding plasmonic metal nanocrystals in semiconductor nanostructures greatly improved the photocatalytic performance of the latter through the LSPR of the former [39, 40]. The concentrated and scattered light of gold nanoparticles contributed to increase the light absorption of CdS, which led to generate more excitons. The heterojunction between Au and CdS would promote charge separation and transfer. In addition, the photocatalytic rate of Au@CdS-CdS nanoflowers was obviously higher than that of Au@CdS thick, though they had the same size and similar spectral absorption range. This might be related to the special morphology of Au@CdS-CdS nanoflowers which had larger specific surface area that means it could provide more reaction sites. At the same mass, the surface area of nanoflowers was more than 4.67 times that of nanospheres. Detailed calculations were shown in the Additional file 1. Therefore, Au@CdS-CdS nanoflowers have excellent photocatalytic activity among all samples.

**Fig. 7:**
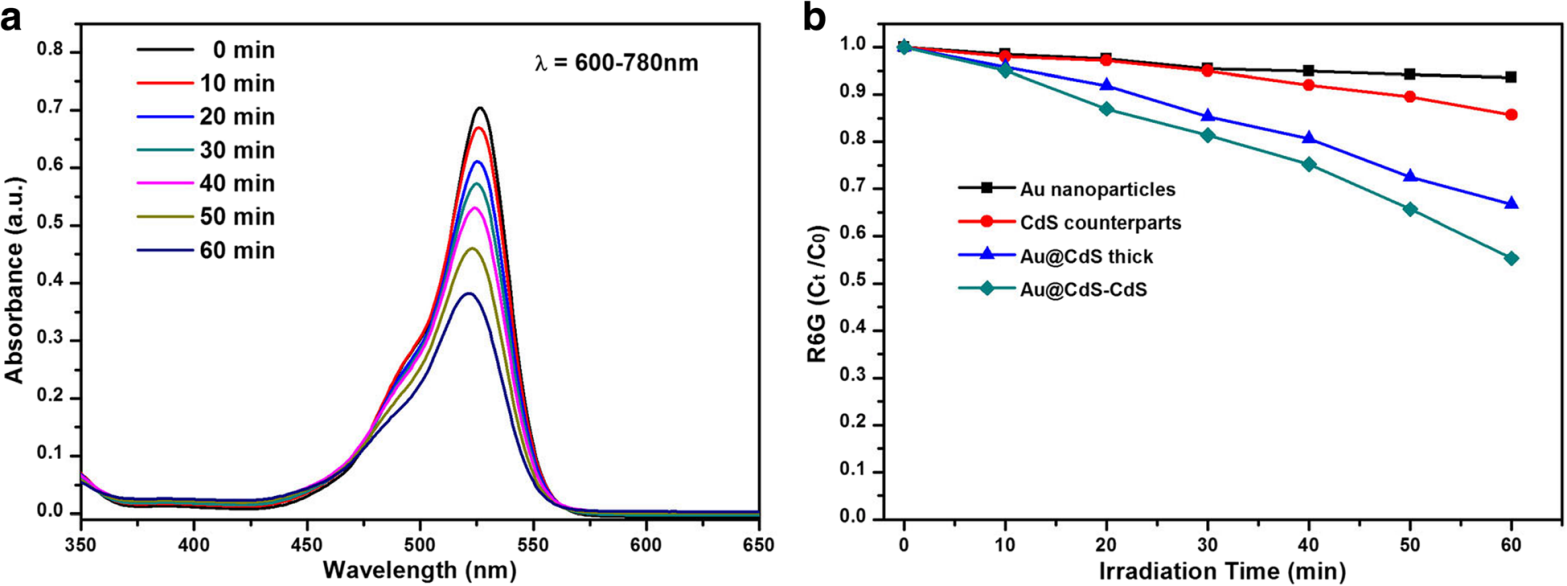
**a** Absorption spectra of R6G solutions at different irradiation times by using Au@CdS-CdS nanoflowers as photocatalyst under irradiation of 600–780 nm. **b** *C*_t_/*C*_0_ versus irradiation time plots for R6G photodegradation in the presence of different photocatalysts

**Fig. 8:**
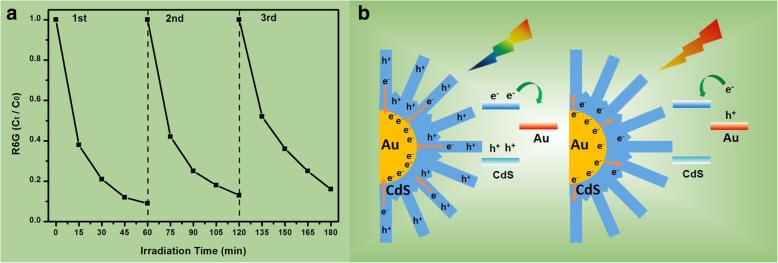
**a** The cycling runs for the photocatalytic degradation of R6G dye over the Au@CdS-CdS nanoflowers under visible light irradiation (400–780 nm). **b** Two charge separation mechanisms for Au@CdS-CdS nanoflowers under 400–780 nm and 600–780 nm irradiation, respectively

There are several primary mechanisms such as near-field mechanism, light scattering, and hot electron injection that can be described for plasmon-enhanced photocatalytic reaction. To test the expanded absorption range of Au@CdS-CdS nanoflowers whether enhanced the photocatalytic activity or not, we conducted photocatalytic tests under the illumination of λ = 600–780 nm. Figure 7a showed the UV-Vis absorption of R6G degraded by Au@CdS-CdS nanoflowers under the illumination of λ = 600–780 nm. The photocatalytic activity for degrading R6G by four photocatalysts were shown in Fig. 7b. After 60-min irradiation, the degradation of Au nanoparticles, CdS counterparts, Au@CdS thick, and Au@CdS-CdS nanoflowers was 9.8%, 15%, 34%, and 45%, respectively. The rate of photodegradation in hybrid nanostructures systems were faster than that of single component crystals. The photocatalytic activity of the hybrid nanostructures were improved by the expanded absorption range because CdS was not excited when incident light above 516 nm. While the SPR absorption peak of Au nanoparticles in Au@CdS-CdS nanoflowers was about 650 nm. Under the illumination of 600–780 nm, hot electrons were generated from Au and would transfer to conduction band of CdS during relaxation [41, 42]. In order to further evaluate the durability and reusability of these photocatalysts, the same sample was centrifuged and redispersed in 20 mL H_2_O containing 10^−5^ M R6G for multiple cycling tests. Figure 8a demonstrated that the efficiency of photodegradation of R6G remains almost unchanged during the three consecutive test cycles, suggesting the high stability of Au@CdS-CdS nanostructures.

Both plasmonic Au and narrow bandgap semiconductor CdS are sensitive to visible light in Au-CdS nanostructure. Thus, two distinct photoinduced charge separation and transfer mechanisms in Au@CdS-CdS nanoflowers are expected to exist depending on the incident excitation energy as shown in Fig. 8b. For a specific case, if only the CdS is excited, the electrons will be transferred from the conduction band of CdS to the Au. Similarly, if only Au is excited, then the electrons will jump from the surface plasmon state of Au to the conduction band of CdS. But the case becomes more complicated when both Au and CdS are excited together. The transfer of electrons in both way are possible, though more probable is the transfer from semiconductor to metal [43–45]. The increased probabilities for charge separation will improve the photocatalytic activity of metal-semiconductor hybrid nanostructures, because separated electrons and holes will have high probabilities for participating in catalytic reactions. Usually, the holes can oxidize the organic contaminants or water molecules to form ^·^OH, and at the same time, the photogenerated electrons are easily captured by the oxidizing substances or other active substances to produce O_2_^·-^. The O_2_^·-^ and ^·^OH have high oxidation efficiencies and excellent chemical activities, which can oxidize the vast majority of organic dyes. The synthesis of hierarchical Au@CdS-CdS nanoflowers will provide a new perspective for rational design and controlled synthesis of hybrid nanostructures with high photocatalytic properties.

## Conclusion

Hierarchical Au@CdS-CdS nanoflowers were successfully prepared by stepwise method. In the obtained nanohybrids, CdS nanorods were epitaxial grown on the surface of Au@CdS CSNs. The Au@CdS-CdS nanoflowers exhibited excellent photocatalytic activity toward the degradation of R6G under the irradiation of λ = 400–780 nm and λ = 600–780 nm, respectively. Hierarchical Au@CdS-CdS nanoflowers had three advantages: first, heterostructure improved the separation and transfer of photogenerated electron-hole pairs; second, the hierarchical structure provide more reaction sites; and third, the expanded absorption range enhanced the ability to capture light. Furthermore, we proposed a possible mechanism for the stepwise synthesis of Au@CdS-CdS nanoflowers, which could be used to prepare other metal-semiconductor nanohybrids with complex morphology for future clean energy and environmental restoration fields.

## Additional File

Additional file 1: Calculation of Surface Area of Samples. **Figure S1.** Nanostructure model of Au nanoparticles, Au@CdS CSNs, Au@CdS-CdS nanoflowers, respectively. (DOCX 85 kb)

## Abbreviations

CSNs: Core-shell nanoprticles
Cys: l-cysteine
Cys/Cd^2+^: l-cysteine-Cd^2+^
EDS: Energy dispersive spectrometer
En: Ethylenediamine
FE-SEM: Field emission scanning electron microscope
HR-TEM: High-resolution transmission electron microscope
NPs: Nanoparticles
PL: Photoluminescence
TEM: Transmission electron microscope
UV-Vis: Ultraviolet-visible
XRD: X-ray diffraction
