# Supplementary material for: Stepwise Synthesis of Au@CdS-CdS Nanoflowers and Their Enhanced Photocatalytic Properties
Source: Nanoscale Res Lett. 2019 Apr 29;14:148. doi: 10.1186/s11671-019-2977-z (PMC6488634; doi:10.1186/s11671-019-2977-z)
Supplement: Supplementary file 1 — Calculation of Surface Area of Samples. Figure S1. Nanostructure model of Au nanoparticles, Au@CdS CSNs, Au@CdS-CdS nanoflowers, respectively. (DOCX 85 kb) [file 11671_2019_2977_MOESM1_ESM.docx]

**Additional file 1**

**Stepwise synthesis of Au@CdS-CdS nanoflowers and their enhanced photocatalytic properties**

**Liwei Wang, Zhe Liu, Junhe Han, Ruoping Li^^[[1]](#footnote-1)^*^ and Mingju Huang^*^**

**Calculation of Surface Area of Samples**


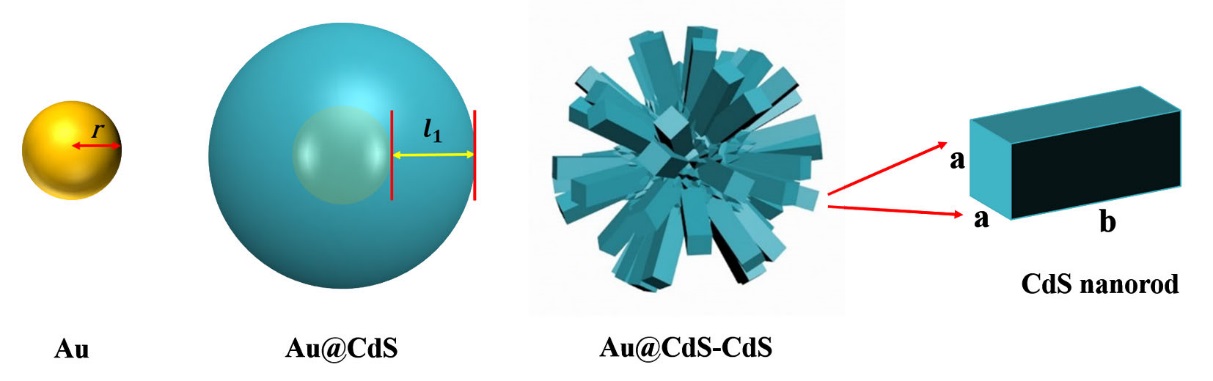


Figure S1 Nanostructure model of Au nanoparticles, Au@CdS CSNs, Au@CdS-CdS nanoflowers, respectively.

$$S_{1}={4\left( r+l_{1} \right)}^{2}$$

$$S_{2}={n(a}^{2}+4ab)$$

$$S_{3}={4(r+l_{2})}^{2}$$

$$n={S_{3}}/{a^{2}}$$

$${S_{2}}/{S_{1}}=2.39$$

where the shape of both the Au nanoparticle and Au@CdS core-shell nanoparticle are assumed spherical, it is also assumed that the CdS nanorods completely cover the surface of the Au@CdS core-shell nanoparticles. $\boldsymbol{S}_{\boldsymbol{1}}$ **,** $\boldsymbol{S}_{\boldsymbol{2}}$ and $\boldsymbol{S}_{\boldsymbol{3}}$ are the surface areas of Au@CdS thick, Au@CdS-CdS nanoflowers and Au@CdS thin, respectively. *r* is the radius of the gold nanoparticle (~25nm), $\boldsymbol{l}_{\boldsymbol{1}}$ and $\boldsymbol{l}_{\boldsymbol{2}}$ are the CdS shell thicknesses of Au@CdS thick and Au@CdS thin, which are ~50 nm and ~10 nm, respectively. ***a*** and ***b*** are the width and length of the CdS nanorod, which are ~16 nm and ~40 nm, respectively. ***n*** represents the number of CdS nanorods on Au@CdS-CdS nanoflowers

$$V_{Au}=\frac{4r^{3}}{3}$$

$$V_{1}=\frac{4{{[(r+l_{1})}^{3}-r}^{3}]}{3}$$

$$V_{2}=\frac{4{[\left( r+l_{2} \right)^{3}-r}^{3}]}{3}+na^{2}b$$

*V_1_* and *V_2_* are the volumes of CdS in Au@CdS thick and Au@CdS-CdS, respectively.

$$m=\rho V$$

$$m_{1}=\rho_{Au}V_{Au}+\rho_{CdS}V_{1}$$

$$m_{2}=\rho_{Au}V_{Au}+\rho_{CdS}V_{2}$$

$${m_{1}}/{m_{2}=}{N_{2}}/{N_{1}=1.98}$$

$m_{1}$ and $m_{2}$ are the masses of Au@CdS thick and Au@CdS-CdS nanoflower, respectively. $\rho_{Au}=19.32g/{{cm}^{3}}$, $\rho_{CdS}=4.82g/{{cm}^{3}}$.$N_{1}$ and $N_{2}$ are the number of Au@CdS thick and Au@CdS-CdS nanoflowers, respectively, of the same mass.

$${A_{2}}/{A_{1}}={N_{2}S_{2}}/{N_{1}S_{1}}=4.67$$

$A_{1}$ and $A_{2}$ are the total surface area of Au@CdS thick and Au@CdS-CdS nanoflowers when the mass is the same, respectively.

1. * Correspondence: lrpmm@henu.edu.cn; hmingju@163.com

   School of Physics and Electronics, Henan University, Kaifeng 475004, People’s Republic of China [↑](#footnote-ref-1)
